# Cleaning Efficacy of Root Canal Irrigation with Positive and Negative Pressure System 

**DOI:** 10.22037/iej.v13i3.20875

**Published:** 2018

**Authors:** Ira Widjiastuti, Dani Rudyanto, Tamara Yuanita, Taufan Bramantoro, Wawan Aries Widodo

**Affiliations:** a * Department of Conservative Dentistry, Faculty of Dental Medicine, Universitas Airlangga, Indonesia; *; b * Resident of Conservative Dentistry, Faculty of Dental Medicine, Universitas Airlangga, Indonesia; *; c * Department of Public Health, Faculty of Dental Medicine, Universitas Airlangga, Indonesia; *; d * Mechanical Engineering Department, Institut Teknologi Sepuluh Nopember, Indonesia*

**Keywords:** Computational Fluid Dynamics, Negative Pressure Irrigation, Positive Pressure Irrigation, Root Canal Irrigants, Smear Layer

## Abstract

**Introduction:**

The purpose of this study was to analyze the differences between irrigant replacement in the positive and negative pressure irrigation systems regarding root canal cleaning efficacy.

**Methods and Materials:**

A total of 27 extracted single-root mandibular premolars with 18-20 mm root canal length were decoronated and equally divided into three groups (*n*=9) based on the irrigation system used: positive irrigation with side-vented needle as the control group (C), positive irrigation with an open-ended needle as the first group (T1) and negative irrigation as the second group (T2). The root canals were irrigated with 2.5% NaOCl between each instrumentation, followed by a final irrigation with 5 mL of sterile distilled water. The irrigation replacements were monitored by means of computational fluid dynamic (CFD), while a scanning electrone microscope (SEM) was used to observe the smear layers and plug evaluations after the teeth had been sectioned longitudinally and buccolingually halves subsequently cut in apical third area. The result was analyzed using the Kruskal Wallis, Mann Whitney and Spearman correlation tests. The level of significance was set at 0.05.

**Result:**

Irrigant replacement in the negative pressure irrigation system tends to produce a greater effect in reaching the apical end compared to in the positive pressure irrigation system. This resulted in significantly superior smear layer removal in the apical third area (*P*<0.05).

**Conclusion:**

The irrigation solution exchange of the negative pressure irrigation system is more capable of reaching the apical end compared to the positive pressure irrigation system, resulting in a higher sanitation level in the apical third of the root canal.

## Introduction

Root canal treatment is performed to eradicate sources of irritation in the root canal and periapical tissues [1]. The goal of root canal treatment is the total elimination of bacteria in infected root canals to prevent re-infection during and after treatment and restoration of healthy periapical tissue [2-5]. To achieve a successful endodontic treatment, chemo-mechanical preparation using the appropriate selection of instrument and irrigation technique is required to successfully reach along the working length and eliminate the smear layer and microbes from the root canal [6-10].

A conventional root canal irrigation system is generally a system for the slow delivery of an irrigation solution into the root canal by means of a syringe [11]. Conventional irrigation with positive pressure system using needle instrument is a standard procedure but found to be inadequate to reach the apical third of root canal and the apical with difficult anatomical features. Therefore, new instruments incorporated with different irrigation system are developed through several researches which confirmed that there was an increase in smear layer elimination efficacy after the usage of sonic, laser and negative pressure irrigation systems, with the latter having the best results [12]. 

Negative pressure irrigation system is a system on which the irrigation needle and suction co-exist, enabling the simultaneous used of suction when delivering the irrigation solution *via* the syringe. This might be regarded as more effective and safer means of delivering the irrigation solution to the apical area so that it can penetrate inaccessible parts such as isthmii, fins and irregular root canals [13-16]. In this system, there was an increase of flow rate resulting in greater liquid volume in apical area to reach the entire working length and improve the sanitation results [17, 18].

Different pressure used in the irrigation process will produce different fluid dynamics inside the root canal system, which will affect the debridement result [15]. In order to identify the differences between the irrigation solution exchange in the positive and negative pressure irrigation systems in relation to the hygiene of the root canals, this research was conducted. Computational Fluid Dynamic (CFD) was used to observe the fluid dynamics simulation, while scanning electron microscope (SEM) was used to evaluate the presence of smear layer on the root canal surface.

## Materials and Methods

In this study, 27 extracted first mandibular premolar teeth were chosen on the basis of the following criteria: average length of 20±2 mm, single root canal, with a perfectly closed root tip and devoid of root defects. The sample preparation begins with soaking the sample in normal saline solution, prior to its division into three groups based on the irrigation system used (*n*=9): positive pressure irrigation system with a closed-ended (side-vented) needle as the control group (C), positive-pressure irrigation system with an open-ended needle as the first treatment group (T1) and negative pressure irrigation system as the second treatment group (T2). 

The samples were prepared by opening the pulp roof using a high-speed endo access bur (Dentsply Maillefer, Ballaigues, Switzerland). The length of samples was measured using #8 and 10 C-Pilot files (VDW GmbH, Munchen, Germany). Thereafter, a glide path was conducted by the sequential use of #8, 10, and 15 files, then continued to root canal preparation with ProTaper Next (Dentsply Maillefer, Ballaigues, Switzerland) up to X3 and irrigated according to the irrigation system used in each group.

**Table 1:** The mean (SD) of distance between the apical end and the peak of the irrigation solution discharged from the irrigation needle

**Group**	**Mean(SD)**
**C**	2.209(0.001)^a^
**T1**	0.441(0.005)^b^
**T2**	0.068(0.015)^c^

a,b,c *Different superscript denotes the significant difference (P<0.05)*

The irrigation was conducted using a Vpro Endo Save (Vista Dental, South St, Racine, USA) negative pressure irrigation device for the T2 group and modified negative pressure irrigation device for the C and T1 groups. The modification was effected by disconnecting the suction and replacing the original needles with open-ended (Ultradent, Utah, USA) and close-ended (side-vented) C-K Endo (CK Dental Ind.Co.Ltd, Korea) irrigation needles. The device incorporated a stopper inside the syringe to control the thumb pressure in order to ensure delivery of 0.2 mL of irrigation solution with each depression of the plunger. The root canals were instrumented and irrigated with 2.5% NaOCl between each instrumentation, followed by final irrigation with 5 mL sterile aquadest. The root canal was then dried using paper points (Dentsply Maillefer, Ballaigues, Switzerland).

A deep cut was made on the buccal and the lingual part of the prepared and irrigated teeth using an NTI Flex disc bur (Kerr, West Collins, USA) to approximately one third of the tooth prior to be vertically split into two parts with a chisel along the buccal-lingual line. The split teeth were then cut at the third area of apical using the bur disc.

The samples were observed using a SEM (Evo MA10, Carl Zeiss, Oberkochen, Germany) under 2500× magnification. The root canal sanitation score was obtained as follows: *score 1*, there was no smear layer in the root canal wall and the dentin tubuli were all open; *score 2*, there was a thin smear layer and some dentin tubuli were open; *score 3*, there was a homogeneous smear layer covering the wall of the root canal, there was no smear layer on the surface of the root canal and most of the dentin tubes were covered with smear plug; *score 4*, a homogeneous smear layer covered all the walls of the root canal, there was no open dentin tubule; *score 5*, there was a thick smear layer covering the entire wall of the root canal [19].

**Table 2:** The score of root canal sanitation in each group

**Group**	**Root canal score**	**Frequency (%)**	***P-value***
**C**	Score 0	0%	0.001*
	Score 1	0%	
	Score 2	0%	
	Score 3	11.11%	
	Score 4	88.89%	
**T1**	Score 0	0%	
	Score 1	0%	
	Score 2	0%	
	Score 3	77.78%	
	Score 4	22.22%	
**T2**	Score 0	0%	
	Score 1	0%	
	Score 2	66.67%	
	Score 3	33.33%	
	Score 4	0%	

* *Significant difference at P<0.05*

**Figure 1:**
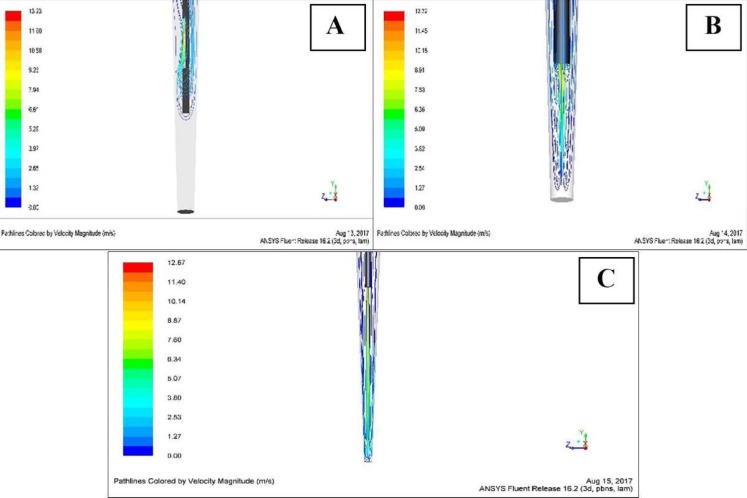
The exchange of irrigation solution in apical third area through CFD simulation. *A)* Positive pressure irrigation system using side-vented needles (C); *B)* Positive pressure irrigation system using open-ended needles (T1); *C)* Negative pressure irrigation system (T2)

**Figure 2:**
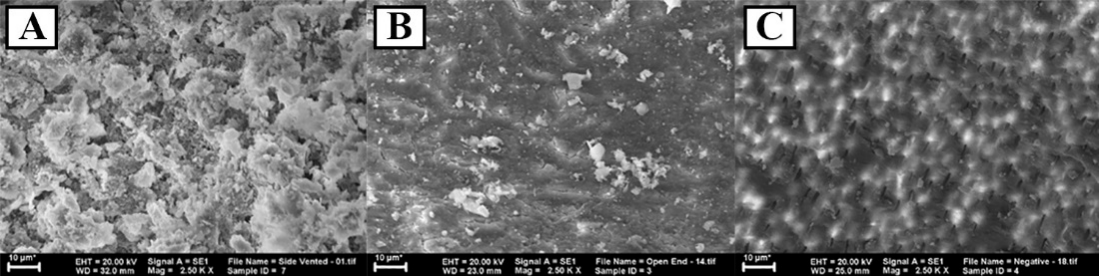
The sanitation of a root canal in a third area of apical through SEM observation under 2500× magnification. *A)* Irrigated with a positive pressure irrigation system using side-vented needles (C); *B)* Irrigated with a positive pressure irrigation system using open-ended needles (T1); *C)* Irrigated with a negative pressure irrigation system (T2)

All statistical tests were performed using R statistical software version 3.4.0 and the test was significant when a *P*<0.05 value was obtained. A normality test result in the irrigation solution exchange data indicates that the exchange rate of the irrigation solution had abnormal data distribution, and in order to compare the result between the groups, the Kruskal-Wallis and Mann-Whitney tests were used. In order to compare the respective SEM scores of the groups, the Kruskal-Wallis and Mann-Whitney tests were also used. In this study, the correlation between the exchanges of irrigation solution with SEM scores using a Spearman correlation test was also analyzed. The level of significance was set at 0.05.

## Results

In this study, the exchange of irrigation solution was performed through CFD simulation (Table 1 and Figure 1), while root canal sanitation observation was measured by means of SEM (Table 2 and Figure 2).

The distance between the apical end and the peak of the irrigation solution between the three groups was significantly different (*P*<0.05). The longest was found in the C group and it was significantly longer compared to T1 group (*P*=0.00) and T2 group (*P*=0.00), while the shortest was encountered in the T2 group which was also significantly shorter compared to T1 group (*P*=0.00) (Table 1).

The result of SEM evaluation of each group could be seen on Table 2. The cleanest sanitation score recorded in T2 group, while the highest number of smear layer presence was found in group C. There were significant differences (*P*=0.001) in the results of the Kruskal-Wallis comparative test between groups and further analysis using Mann-Whitney also showed a significant difference with the value of *P*<0.05. Figure 2 shows the sanitation of the root canal in apical third area through SEM observation in each group.

A Spearman correlation test was conducted to discover the correlation between the irrigation solution exchange value and the sanitation of the root canal which confirmed a significant strong linear correlation (*r*=0.759; *P*=0.001).

## Discussion

In this study, the mean value of the distance between the apical-end and the peak of the irrigation solution discharged from the irrigation needle outlet in group C was significantly greater when compared to the T1 group with positive pressure irrigation systems using open-ended needles and the T2 group with negative pressure irrigation. The longest distance was found in group C due to the shape of the closed-ended needle (side vented) which caused the irrigation solution in the needle to flow in a straight line before encountering the end of the closed-ended needle. It then back-flowed and formed a vortex, a relatively stable circular rapid movement of the fluid, in the needle. The vortex formation caused a slower irrigation solution flow rate, before it was discharged from side-vented outlet and formed several narrow high-speed jets of fluid flowing out of a small diameter hole. The flow of an irrigation solution that strikes the root canal wall would also produce a vortex causing most of the irrigation solution to flow back to the coronal orifice and significantly decrease the irrigation solution flow rate to the apical area [15].

The decrease in the irrigation solution flow rate in the side-vented irrigation system and the presence of an apical vapor lock severely limited the movement of the irrigation solution around the tip of the needle, resulting in a relatively smaller irrigated area and slower penetration of the apical area compared to the open-ended needle. This condition caused limitations on the exchange of irrigation solution in the apical area, resulting in poorer sanitation of the root canal in apical third area compared to the T1 and T2 groups [15]. 

The mean value of the distance between the apical-end and the peak of the irrigation solution in T1 group was 0.441 mm which confirmed that the irrigation solution exchange in the positive pressure irrigation system using open-ended needles was unable to reach the apical. The design of the open-ended needles allowed the jets to flow further into the apical area compared to the jets formed in the side-vented needle. However, this design can also enable direct interaction between the irrigation solution and the trapped air in the apical area to form air bubbles, a phenomenon known as the apical vapor lock. The presence of the apical vapor lock will gradually decrease the jet flow rate from the needle tip before flowing back to the coronal orifice [15]. The inability of irrigation solution exchanges to reach the apical resulted in an ineffective debridement process in the apical area [11, 14].

The shortest distance between the apical-end and the peak of the irrigation solution was 0.068 mm, which was found in the T2 group using the negative pressure irrigation system. This indicates that the negative pressure irrigation system is capable of effectively delivering irrigation solution from the tip of the irrigation needle to the apical end. The negative pressure irrigation system allowed both irrigation solution deliveries through the needle and negative pressure formation through suction at the same time. Based on the study of Fukumoto *et al.* [20], the amount of negative pressure through suction connected with the dental unit is approximately -20 kP. The negative pressure formed a vacuumed area (< 1.01325 × 105 Pa) in the root canal and eliminated the possibility of apical vapor lock formation [21]. The existence of a vacuum in the root canal permits a constant and continuous flow of the irrigation solution to almost reach the apical end, resulting in a greater volume of irrigation solution being delivered to the root canal compared to the positive pressure irrigation system and improving the effectiveness of smear layer elimination [3, 21, 22]. Moreno *et al.* [17] stated that negative pressure irrigation system could increase irrigation solution volume and flow rate in the third apical area; as a consequence, it could reach the entire working length and improve smear layer removal. This ameliorates the sanitation level of the root canal in a third area of apical compared to the T1 group. This result is in accordance with the findings of Fukumoto *et al*. [20] and Mendonca *et al.* [22], which stated that the effectiveness of the smear layer cleaning process on the negative pressure irrigation system is preferable to that of the positive pressure irrigation system. Some studies, which also used SEM analysis to evaluate the effectiveness of smear layer removal, has a consistent result with this study. It suggested that Endovac system were more effective in removing smear layer compared to endo activator [23, 24].

The result of the correlation test showed that irrigation solution exchange has a strong linear relationship with the sanitation of the root canal. The shorter the distance between the apical-end and the peak of the irrigation solution, the greater the level of root canal sanitation in a third area of apical based on the SEM score. These results were in accordance with the previous study conducted by Versiani *et al.* [25] using micro-CT imaging analysis, which concluded that negative pressure irrigation system could remove more hard tissue debris compared to positive pressure irrigation system. In addition, acccording to Azim *et al.* [26], negative pressure irrigation system could also prevent irrigant extrusion.

## Conclusion

The irrigation solution exchange of the negative pressure irrigation system is more capable of reaching the apical end compared to the positive pressure irrigation system, resulting in a higher sanitation level in a third apical area of the root canal.

## Conflict of Interest:

‘None declared’.
